# HLA class II donor specific antibodies are associated with graft cirrhosis after liver transplant independent of the mean fluorescence intensity level

**DOI:** 10.1186/s12876-020-01427-4

**Published:** 2020-08-27

**Authors:** Katharina Willuweit, Alexandra Frey, Lisa Bieniek, Andreas Heinold, Matthias Büchter, Peter A. Horn, Heiner Wedemeyer, Kerstin Herzer

**Affiliations:** 1 Department of Gastroenterology and Hepatology, University Hospital Essen, University of Duisburg-Essen, Hufelandstrasse 55, 45122 Essen, Germany; 2 Institute for Transfusion Medicine, University Hospital Essen, University of Duisburg-Essen, Essen, Germany; 3 Department of Internal Medicine, St. Nikolaus Stiftshospital, Andernach Teaching Hospital, University of Bonn, Andernach, Germany

**Keywords:** Livertransplant, Donor specific antibodies, Complications, Liver cirrhosis, HLA-antibody

## Abstract

**Background:**

The importance of donor-specific antibodies (DSA) after liver transplantation (LT) for graft and patient survival is an ongoing controversy. So far it has not been elucidated when and in how far DSA are harmful for graft and patient survival. Therefore, we had the aim to investigate the association of DSA with complications after LT.

**Methods:**

Data of 430 LT recipients were collected and statistically analyzed. Detection of HLA antibodies (Ab) was performed by Luminex assay.

**Results:**

DSA were detected in 81 patients (18.8%). These were mainly HLA class II Ab (81.5%). HLA class II Ab show a higher MFI (median: 5.300) compared to HLA class I Ab (median: 2.300). There is no association between MFI levels and development of complications after LT. However, cirrhosis occurred significantly more often in DSA positive patients (18%) than in patients without detectable DSA (9%, *P* = 0.027). All DSA positive patients with cirrhosis of the graft showed HLA class II antibodies (OR: 3.028; 95% CI: 1.51–6.075; *P* = 0.002).

**Conclusion:**

Occurrence of HLA class II DSA after LT is associated with graft cirrhosis and may indicate a higher risk to develop graft damage independent on MFI and requires an individualized risk management.

## Background

While DSA are established as risk factor for humoral rejections and lower graft and patient survival after kidney transplant, the role of DSA after LT and their contribution to graft failure and complications is still not clarified and debates remain controversial. Some studies suggested that DSA are harmful after LT and reported associations with acute antibody-mediated rejection (AMR), biliary strictures and long-term complications, such as graft fibrosis and chronic rejection [1–3]. However, some of the observations may be explained by distinct circumstances and require validation in independent cohorts. We could recently show a higher prevalence of DSA in patients with autoimmune liver diseases (*primary sclerosing cholangitis* [PSC], primary biliary cirrhosis [PBC], and autoimmunhepatitis [AIH]) as underlying liver disease for LT. On the other hand distinct immunosuppressive drugs may influence the development of DSA as in out cohort an mTor inhibitor based immunosuppressive regimen reduced the risk to develop DSA [4].

Protection from DSA damage may be provided by the clearing effect of the liver as an organ with the capability to absorb DSA. This “liver tolerance effect” privileges liver transplant patients to require less immunosuppression in the maintenance setting than recipients of other organs and also to be at less risk for episodes of hyperacute rejection [5, 6]. Nevertheless, there is evidence that in some cases DSA also after LT contribute to more complicate courses [7–9].

It is likely that certain associated factors determine whether DSA are harmful and contribute to graft damage. Süsal et al. reported that among renal transplant patients preactivated T cells are necessary for DSA to exert a deleterious effect; among these patients, soluble CD30 was found to be an activation marker [10].

The interest in specific human leukocyte antigen (HLA) classes increased after several studies reported DSA development during AMR episodes. These antibodies (Ab) frequently targeted HLA DQ antigens after renal transplant [11].

The distinct role of class II DSA and the MFI levels in DSA detection assays is not well defined after LT. Some researcher groups studying anti-HLA Abs after LT reported that the DSA associated with complications are usually class II DSA with high mean fluorescence intensity (MFI) levels [6, 12]. However, the relevance of high MFI levels remains debatable, and the clinically meaningful MFI threshold that predicts an increased risk of complications after LT has not been determined.

Thus, the objective of the current study was to investigate the prevalence of DSA among a large cohort of LT patients and to determine the association of complications with HLA classes and MFI levels.

## Methods

### Patients

This study included 430 consecutive LT patients who were participating in regular aftercare at the University Hospital Essen. We screened these patients for the presence of DSA and retrospectively collected demographic data, patient characteristics, serological and clinical data from the patients’ charts for statistical analysis. DSA screening was performed post-transplant and no information about HLA status before transplant was available. The study was conducted in accordance with the Helsinki Declaration of 1975 and was approved by the ethics committee of the University Hospital Essen (AZ 16–6815-BO).

### Antibody detection

HLA Abs were detected with a Luminex-based anti-HLA Ab screening assay (LABScreen Mixed; One Lambda, Canoga Park, CA, USA). Only HLA Abs of positive reacting sera and were subsequently specified with a Luminex single-antigen bead assay (LABScreen Single Antigen; One Lambda). For the LABScreen Mixed assay, a normalized background ratio higher than 3 was considered positive. For the specification of DSA with the LABScreen Single Antigen assay, an MFI value above the threshold of 500 was required. In case of multiple DSA detection the cumulative MFI values were used.

### Data analysis and statistical methods

To assess significant differences between two groups a two-tailed Student’s t-test or Mann-Whitney U-test was used. Statistical significance was analyzed by Fishers exact test or χ^2^-test with Pearson approximation. Independent prognostic markers were determined by multivariate analysis. Therefore a logistic regression model was used. A *P-*value of ≤0.05 was considered to be significant. Statistical analyses were performed using SPSS statistical software (IBM SPSS Statistics for Windows, Version 19; IBM Corporation, Armonk, NY, USA).

## Results

### Baseline characteristics

Patients’ characteristics are outlined in Table 1. Average age at the time of LT was 50 years (range, 1–68 years). Of the 430 patients included in this study, 252 (59%) were male. The most frequent reason for LT was cirrhosis due to chronic hepatitis C virus (HCV) infection (23.7%) or secondary alcoholic steatohepatitis (ASH; 22.1%) (Table 1). The median time from LT to the time of sample collection was 41 months (range, 1–303 months). Donor data were available for 377 patients. We observed no significant differences in prevalence of DSA with regard to donor-recipient sex-mismatch (*P* = 0.813).

**Table 1:** Patient and donor characteristics

Patient characteristics	Patients (n/%)	DSA negative	DSA positive	***P***-value
Total patient numbers	430	349 (81.2)	81 (18.8)	–
Age of recipient, years	50 (1–68)	50 (1–68)	49 (1–63)	0.156
BMI of recipient, kg/m2	24 (10.0–55.0)	24.5 (10.0–55.0)	24.2 (10–32.5)	0.306
Weight of recipient, kg	72 (36–157)	73 (36–157)	70 (43–103)	0.022
MELD	17 (6–40)	17 (6–40)	17.5 (6–40)	0.940
Cold ischemia time, h	7:14 (1:01–20:00)	7:14 (1:01–18:10)	7:14 (1:15–20:00)	0.363
Sex, m/f (%)	252 (59)/178 (41)	211 (60.5)/138 (39.5)	41 (50.6)/40 (49.4)	0.133
**Donor characteristics**				
Total donor numbers	377	–	–	–
Age of donor, years	51 (2–88)	51 (2–88)	49 (3–82)	0.171
BMI of donor, kg/m2	25.0 (11.0–51.0)	25 (11–51)	25 (10.3–36)	0.236
Weight of donor, kg	75 (12–170)	75 (12–160)	75 (17–170)	0.316
Sex D/R: f + f; m + m/f + m; m + f	232 (61.5)/145 (38.5)	194 (83.6)/111 (76.6)	38 (16.4)/34 (23.4)	0.106
**Indication for LT**				
HCV	102 (23.7)	86 (24.6)	16 (19.8)	0.387
ASH	95 (22.1)	87 (24.9)	8 (9.9)	0.003
HCC	91 (21.2)	83 (23.8)	8 (9.9)	0.006
HBV	70 (16.3)	56 (16.0)	14 (17.3)	0.741
NASH	69 (16.0)	57 (16.3)	12 (14.8(	0.867
PSC/PBC/AIH (AIL)	64 (14.9)	46 (13.2)	18 (22.2)	0.055
Acute liver failure	32 (7.4)	23 (6.6)	9 (11.1)	0.163
Other	29 (6.7)	26 (7.4)	3 (3.7)	0.325

Patient characteristics given as median and range and etiology of liver disease (n/%)

*AIH* autoimmune hepatitis, *AIL* autoimmune liver disease, *ASH* alcoholic steatohepatitis, *BMI* body mass index, *D* donor, *DSA* donor specific antibody, *f* female, *HBV* hepatitis B, *HCV* hepatitis C, *LT* liver transplant, *m* male, *MELD* model of end stage liver disease, *NASH* nonalcoholic steatohepatitis, *PBC* Primary biliary cholangitis, *PSC* primary sclerosing cholangitis, *R* recipient

### DSA prevalence and distribution of HLA classes

**Fig. 1:**
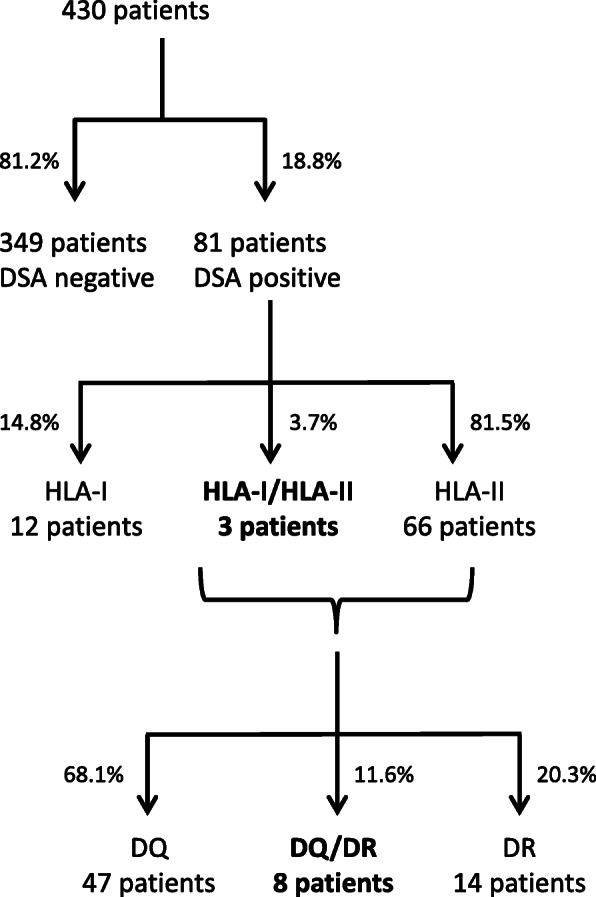
Flow diagram of donor-specific antibody prevalence. A total of 430 patients were included in the analysis. DSA, donor-specific antibody; HLA, human leukocyte antigen

Overall, 81 patients (18.8%) tested positive for DSA. Of these patients, 66 (81.5%) tested positive for anti-HLA class II DSA, 12 (14.8%) for anti-HLA class I DSA (14.8%), and 3 (3.7%) for both anti-HLA class I and class II DSA. DSA were more prevalent among female LT recipients (40/178; 22.5%) than among male recipients (41/252; 16.3%). Of the 81 patients with anti-HLA class II Abs, 47 (68.1%) had antibodies against HLA class II DQ and 14 (20.3%) had antibodies against HLA class II DR; only 8 patients (11.6%) tested positive for both anti-HLA class II DQ and anti-HLA class II DR Abs (Fig. 1).

### Association between complications after LT and DSA status

**Fig. 2:**
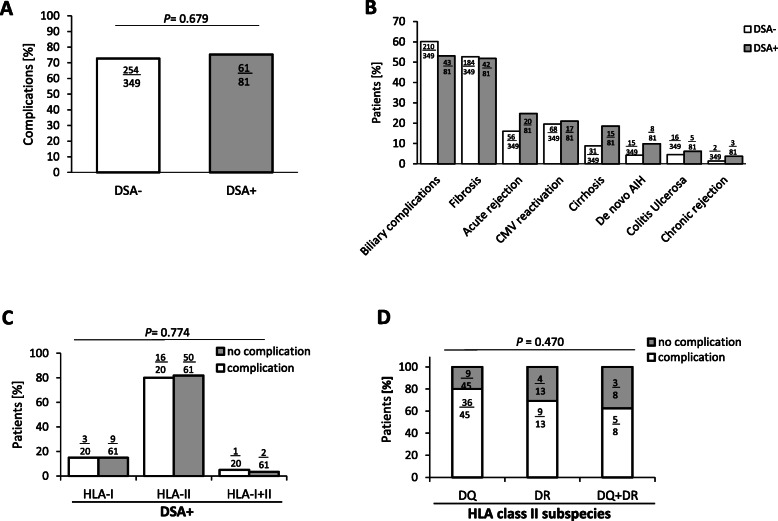
Occurrence of complications in donor-specific antibody (DSA)-negative and DSA+ patients after liver transplant. **a** Occurrence of complications in the two groups. **b** Occurrence of various complications among DSA-positive and DSA-negative patients. **c** Percentage of DSA-positive patients in the various HLA classes with regard to the occurrence of complications. **d** Percentage of complications according to HLA class II antibodies after liver transplant. AIH, autoimmune hepatitis; CMV, cytomegalovirus; DSA, donor-specific antibody

In total, 315 (73.3%) of the 430 patients experienced liver graft complications related to the transplant (Fig. 2a, b). The patient cohort was screened for differences in complication rates with regard to their DSA status. Patients with detectable DSA did not experience LT-associated complications (61/81; 75.3%) more often than did DSA-negative patients (254/349; 72.8%; *P* = 0.679) (Fig. 2a).

The most frequently occurring complications were biliary in nature (253/430; 58.8%). These complications occurred in 43 of the 81 (53%) DSA-positive patients and in 210 of the 349 (60.2%) DSA-negative patients (*P* = 0.261). De novo AIH (dnAIH) tend to occur more often among DSA-positive patients (8/81; 9.8%) than among DSA-negative patients (15/349; 4.3%; *P* = 0.055). In addition, 20 of the 81 (24.7%) DSA-positive patients but only 56 of the 349 (16%) DSA-negative patients experienced biopsy-proven acute rejection (*P* = 0.076).

For our patient cohort, DSA status was associated with the development of cirrhosis. Graft cirrhosis developed in 15 of the 91 (18%) DSA-positive patients but in only 31 of the 349 (9%) DSA-negative patients (*P* = 0.027; Fig. 2b). Multivariate analysis determined that positive DSA status (odds ratio [OR], 2.3; *P* = 0.026) after LT is an independent predictor of recurrent liver cirrhosis and is not dependent on underlying liver disease, time after LT, patients gender and age.

No significant differences between DSA-positive and DSA-negative patients were found with regard to chronic rejection (*P* = 0.176), fibrosis (*P* = 0.805) or cytomegalovirus (CMV) infection (*P* = 0.878).

As a further step, we were interested to determine whether Abs in any HLA class are associated with the occurrence of complications. We found no significant relationship between the prevalence of HLA classes and complications of any sort (*P* = 0.938) (Fig. 2c). Overall, complications occurred among 4 of the 13 patients (30%) who tested positive for HLA class II subgroup DR and among 9 of the 45 patients (20%) who tested positive for HLA class II subgroup DQ (*P* = 0.47). Of the 8 patients who tested positive for both subgroups, 3 (37%) experienced complications (Fig. 2d).

### MFI level is not an indicator of future complications

**Fig. 3:**
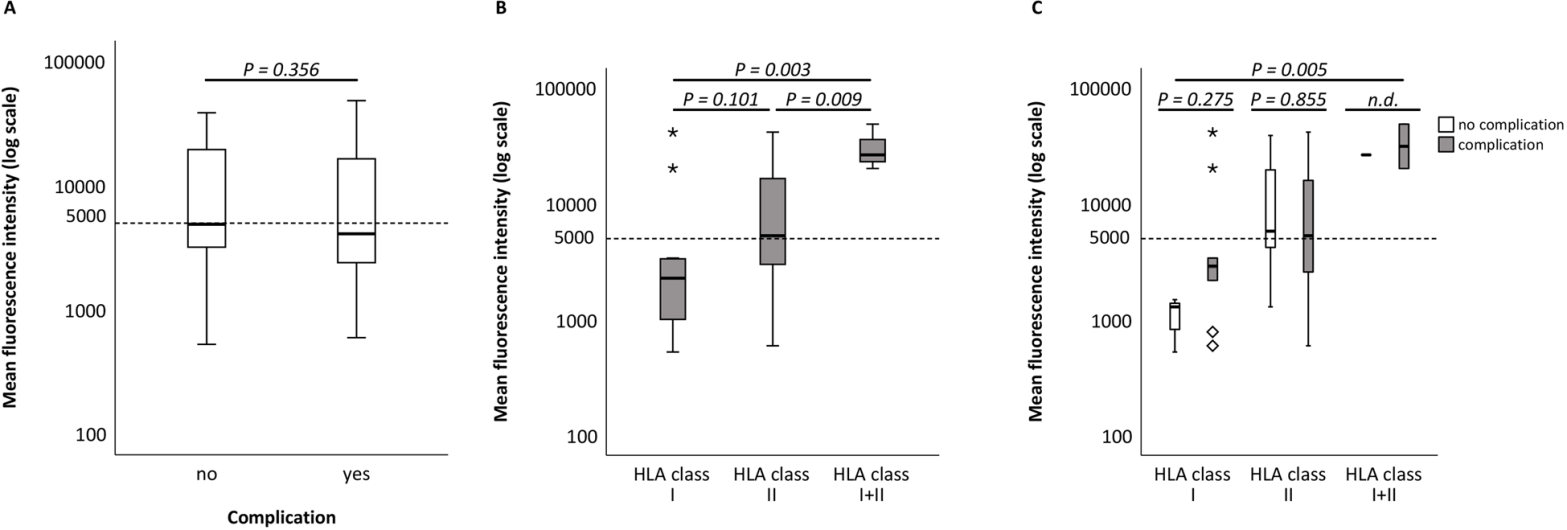
Mean fluorescence intensity levels according to presence of posttransplant complications, according to HLA class, and according to presence of donor-specific antibodies. **a** Occurrence of complications in association with mean fluorescence intensity (MFI) levels. **b** Correlation between HLA classes and MFI levels. Average MFI levels for patients with detectable HLA-I donor-specific antibodies (DSA were lower than 5000; those for patients with detectable HLA-II DSA were approximately 5000 and those for patients testing positive for both HLA-I and HLA-II DSA were higher than 10,000 (*P* = 0.005). **c** Prevalence of complications in association with HLA classes and MFI levels. Within the various HLA classes there were no significant differences in MFI levels between liver transplant patients with and without complications. DSA, donor-specific antibody; HLA, human leukocyte antigen

In our patient cohort, MFI was not associated with the occurrence of complications (*P* = 0.356) (Fig. 3a). For patients testing positive for HLA class II Abs, MFI levels were mainly below 5000 (median, 2300; range, 530–41,300); for patients testing positive for HLA class II Abs, average MFI levels were somewhat higher, at approximately 5000 (median, 5300; range, 600–41,500). Average MFI levels for patients testing positive for both anti-HLA class I Abs and anti-HLA class II Abs were significantly higher, (> 10,000 median, 26,400; range, 20,140-48,600) (*P* = 0.003) (Fig. 3b).

We also addressed the question of whether MFI levels differed between LT patients experiencing complications and those not experiencing complications. All patients testing positive for Abs from each HLA class (HLA- class I, *n* = 12; HLA- class II, *n* = 66; HLA- class I/HLA- class II, *n* = 3) were differentiated with regard to the occurrence of complications. MFI levels were not significantly different between DSA-positive patients with or without complications, nor were they significantly different between patients with Abs from the various HLA subclasses. Of the 12 patients with HLA class I ABs, 3 experienced no complications at a median MFI of 1300; whereas 9 experienced complications at a median MFI of 2900 (*P* = 0.275). Of the 66 patients with HLA-class II Abs, 16 experienced no complications at a median MFI of 5850, whereas 50 experienced complications at a median MFI of 5300 (*P* = 0.855). Only three patients tested positive for both HLA-class I and HLA-class II. Two of them (66.7%) experienced complications at a median MFI of 43,700; the other patient experienced no complications at a median MFI of 26,400 (*P*: n.d.) (Fig. 3c).

### Cirrhosis of the graft tends to develop among DSA-positive patients with HLA class II antibodies

Although we found that HLA classes were not associated with the occurrence of complications in general, we were especially interested in their influence on graft cirrhosis. We described that cirrhosis developed more often among DSA-positive patients (15/81; 18%) than among DSA-negative patients (31/349; 9%; *P* = 0.027) (Fig. 2b).

**Fig. 4:**
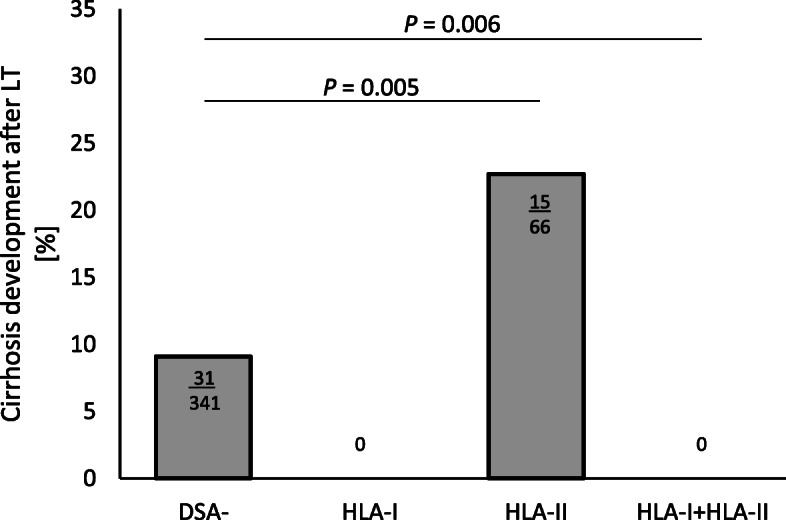
Development of cirrhosis after liver transplant. Frequency of development of cirrhosis presented as percentage of patients testing positive for donor-specific antibodies and those testing negative for such antibodies, separated according to human leukocyte antigen class. DSA, donor-specific antibody; HLA, human leukocyte antigen; LT, liver transplant

In more detail and interestingly, all DSA-positive patients in whom cirrhosis developed tested positive for HLA class II antibodies (OR, 2.94; 95% confidence interval, 1.48–5.83; *P* = 0.006; Fig. 4). Further investigations by multivariate analysis showed that HLA class II–positive DSA status was an independent predictor of recurrent cirrhosis of the graft, independent of the patient’s underlying disease, such as HCV (OR, 2.51; *P* = 0.009). We found no correlation between the development of cirrhosis and the presence of HLA class II DQ or DR (*P* = 0.618).

## Discussion

The present study investigated whether certain DSA classes or the MFI of DSA affects the occurrence of any transplant-related complications after LT.

We observed a significantly higher incidence of graft cirrhosis among DSA-positive patients. Furthermore, HLA class II DSA could be differentiated as statistically significant association for graft cirrhosis independent on the MFI level.

Our results agree with those of other reports about complications after LT in association with DSA. A large retrospective study by Kaneku et al. found that 61 of 749 patients (8.1%) exhibited de novo DSA (dnDSA) within one year after LT. The 5-year graft and patient survival rates were significantly lower for these patients than for those without detectable DSA [13]. Moreover, recent data have shown that LT recipients with preformed or dnDSA are at higher risk of progression of fibrosis [14].

In our cohort, biliary complications were the primary type of complication after LT but were not significantly correlated with the occurrence of DSA. In contrast, others have reported the presence of DSA in association with bile duct complications after LT [3, 15]. A trial by Iacob et al. involving 162 patients examined the clinical risk factors for the occurrence of post-LT ischemia-like biliary lesions and biliary anastomotic strictures (AS). AS developed in 9 of the 40 patients (22.5%) who tested positive for HLA class II DSA and in 7 of the 85 patients without detectable DSA (8.2%) [3]. The bile duct tree has arterial-only circulation and is therefore possibly more sensitive to harmful factors than are hepatocytes, which results in “liver resistance” to Ab-mediated injury. This resistance mechanism includes the large size of the sinusoidal microvascular bed of the liver, the secretion of soluble HLA antigens, Kupffer cell phagocytosis of immune complexes, and the dual afferent circulation of the liver (portal vein and hepatic artery) [16, 17].

DSA have been found to be associated with severe acute and chronic rejection. Persistent preformed DSA can cause severe early humoral rejection and graft failure, chronic rejection, and accelerated progression of fibrosis [15, 18]. A study by Del Bello involved 152 patients without preformed antibodies who were regularly tested for DSA within 12 months after LT and annually thereafter. Over a 34-month study period, dnDSA developed in 21 of 152 patients (14%), and 9 (43%) of these exhibited positive C4d staining with clinical signs of AMR [6, 12]. In our cohort, patients with detectable DSA experienced acute rejection episodes more often than did DSA-negative patients (24.7% vs. 16%; *P* = 0.076).

There is controversy about the relevance of specific HLA loci. In cases of acute rejection, HLA class I antigen expression is increased and HLA class II antigens, especially DR and DP but also DQ, are overexpressed on endothelial cells and among patients with bile duct complications [19].

In addition, Balan et al. found that the presence of HLA-DR3 and HLA-DR4 is associated with AIH after LT. In this context, it is probable that an HLA-DR3 or an HLA-DR4 mismatch is a risk factor for recurrent AIH and PBC [20]. The pathogenesis remains unclear. Some studies suggest that recipient memory T cells play a role in regulating the process by recognizing autoantigenic peptides presented by mismatch donor HLA molecules in the allograft [21].

The evaluation of MFI levels and HLA loci is not standardized and varies across studies, which may analyze MFI levels cumulatively, medially, or separately.

We observed that MFI levels were higher for HLA class II Abs than for HLA class I Abs, although MFI was not associated with the occurrence of complications after LT. O’Leary et al. reported a correlation between preformed DSA with MFI levels higher than 5000 and a higher prevalence of chronic rejection episodes (30% vs 8%; *P* = 0.04). Preformed persistent and de novo HLA class II DSA were more prevalent and were found in association with higher MFI levels among patients exhibiting episodes of chronic rejection [2]. In 2013 O’Leary et al. reported that 32 of 60 LT patients (53%) with allograft injury early after transplant had detectable DSA. This analysis also demonstrates that high MFI levels are predictive for persistence of preformed class II DSA [22]. In Kidney transplant, DSA are a known risk factor for allograft injury, and finding donors whose anti-HLA Abs match those of the recipient is a challenging task. In a study involving 189 patients after renal transplantation, Gloor et al. compared patients with a positive crossmatch (*n* = 119) with a control group (*n* = 70) and examined the correlation of MFI levels with AMR episodes. They reported that high levels of DSA (> 10,000) at baseline (before initiation of desensitization) were associated with a higher risk of AMR (DSA-positive patients, 34/66, 52%; control group, 13/51, 26%; *P* = 0.003) [23].

As a matter of fact, some studies have found that the liver can accommodate DSA when MFI levels are low and that the presence of high-MFI Abs can lead to graft dysfunction [2].

Wozniak et al. reported that HLA class II Abs exert a stronger negative impact than HLA class I Abs. In particular, HLA-DQ Abs are associated with de novo autoimmune hepatitis (dnAIH), late acute cellular rejection, and chronic rejection after pediatric LT [24]. O^.^’Leary et al. found that 32 of 60 LT patients (53%) exhibited unexplained early (< 90 days after LT) preformed DSA [25]. This study showed that patients who experienced allograft loss caused by AMR tested positive for class I and II DSA and exhibited higher MFI levels. A possible explanation is that high-MFI class I DSA overwhelm potential liver resistance mechanisms, such as class I antigen expression in the microvasculature of the liver, resulting in rapid allograft failure. Our findings agree with those of other studies and indicate that HLA class II Abs are more prevalent than class I Abs and are characterized by higher MFI levels [2]. Even so, we found no significant correlation between specific HLA loci and MFI levels. It should be noted that a possible influence on the results of our study and those of others is the length of time between LT and detection of DSA. It is known that preformed DSA usually disappear within one year after LT and that class II Abs persist longer than class I Abs [2]. These facts may explain why, among patients with complicated courses, DSA are not always detectable even though complications such as graft cirrhosis are present. Thus, DSA can contribute to graft cirrhosis but are not always detectable when the protracted cirrhosis process is first clinically apparent.

In contrast, not all DSA-positive patients experience complications after LT, perhaps because the liver, unlike the kidney, has a regenerative ability and a wide vascular surface, as well as decreased complement activation and HLA class II expression in its microvasculature, and Kupffer cells that clear alloantibodies [6, 26]. The liver’s ability to absorb HLA class I Abs and the limited expression of HLA class II antigens on the hepatic microvasculature contribute to the preponderance of HLA class II Abs after LT [26]. Taner et al. performed a prospective trial involving 90 LT patients and found that DSA levels decreased within the first week after LT among 90% of patients [27]. Even when Abs persisted, there were no marked differences in patient and graft survival between DSA-positive and DSA-negative patients. Castillo-Rama et al [28] determined the number of mismatches between donors and recipients and analyzed 5-year survival rates after LT. For 853 patients, no significant difference in survival was associated with the number of mismatches.

Last, but not least, because complications do not develop among all patients with detectable DSA after LT, it is possible that cofactors may be necessary for DSA to become harmful. Thus, OʼLeary et al. [14] studied whether non-HLA autoantibodies, such as those against angiotensin II type 1 receptor and endothelin type A receptor, cause allograft injury by functional non-HLA Abs. That study involved 1269 LT patients and found that a combination of preformed non-HLA autoantibodies and HLA DSA was associated with a higher risk of death. Biopsy samples containing de novo non-HLA autoantibodies exhibited a different sinusoidal C4d staining pattern than did HLA DSA (71% vs 3%; *P* = 0.001). Furthermore, C4d-positive staining showed that sinusoidal endothelial cell activation and stellate cell activation were higher among patients with non-HLA autoantibodies.

Obviously, DSA influence the course after LT, but it is likely that the presence of cofactors will influence whether DSA cause graft damage. Convincing evidence indicates that MFI influences the occurrence of complications and that low-MFI DSA should be taken seriously. Because several risk factors are known to influence DSA development, patients, especially those at high risk of DSA and those with preformed DSA may benefit from closer monitoring and intensified screening when dysfunction or complications occur.

However, some limitations should be noted. The current study is retrospective. Therefore, C4d staining was not performed when acute rejection episodes occurred. Furthermore, because screening for DSA was performed once independently at the time of LT, the length of time from LT to Ab screening ranges from 1 month to 29 years. Our analysis cannot determine whether the measured Abs were preformed or developed de novo after LT. These limitations are currently being addressed by a study collecting data prospectively from early after LT through maintenance aftercare. Patient recruitment and analysis of data from that cohort are currently under way.

## Conclusion

Patients with detectable DSA, especially anti-HLA class II Abs, can be at higher risk of complications after LT, in particular cirrhosis of the graft. Screening of anti-HLA Abs may be useful in the early detection of high-risk patients who could benefit from closer surveillance and higher trough levels of the immunosuppressive regimen. However, patients other than those with high levels of MFI Abs are also at risk of complications after LT. Therefore, it is likely that there are additional factors that make an Ab detrimental. Until more evidence has been gathered, we suggest a multi-track strategy in which the first step is detecting potentially high-risk patients on the basis of the underlying disease and additional sensitizing events, such as pregnancy, blood transfusions, and repeat transplant. Because to date no therapeutic procedures have proven to be very efficient, prophylaxis against DSA development is very important, and maintaining above-average trough levels of immunosuppressants should be considered for at-risk patients.

## Data Availability

The datasets used and analyzed during the current study are available from the corresponding author on reasonable request.

## Abbreviations

Ab: Antibody
AIH: Autoimmune hepatitis
AMR: Antibody-mediated rejection
AS: Anastomotic stricture
ASH: Alcoholic steatohepatitis
CMV: Cytomegalovirus
dnAIH: de novo autoimmune hepatitis
dnDSA: de novo donor-specific antibody
DSA: Donor-specific antibody
HCV: Hepatitis C virus
HLA: Human leukocyte antigen
LT: Liver transplant
MFI: Mean fluorescence intensity
mTOR: mammalian target of rapamycin
OR: Odds ratio
PBC: Primary biliary cirrhosis
PSC: Primary sclerosing cholangitis

## References

[1] O'Leary JG, Kaneku H, Banuelos N, Jennings LW, Klintmalm GB, Terasaki PI (2015). Impact of IgG3 subclass and C1q-fixing donor-specific HLA alloantibodies on rejection and survival in liver transplantation. Am J Transplant Off J Am Soc Transplant Am Soc Transplant Surg.

[2] O'Leary JG, Kaneku H, Susskind BM, Jennings LW, Neri MA, Davis GL (2011). High mean fluorescence intensity donor-specific anti-HLA antibodies associated with chronic rejection Postliver transplant. Am J Transplant Off J Am Soc Transplant Am Soc Transplant Surg.

[3] Iacob S, Cicinnati VR, Dechene A, Lindemann M, Heinemann FM, Rebmann V (2012). Genetic, immunological and clinical risk factors for biliary strictures following liver transplantation. Liver Int.

[4] Willuweit K, Heinold A, Rashidi-Alavijeh J, Heinemann FM, Horn PA, Paul A, et al. Immunosuppression with mTOR inhibitors prevents the development of donor-specific antibodies after liver transplant. Clin Transplant. 2017;31(6):e12974. 10.1111/ctr.12974 28345271

[5] Jordan SC, Vo AA (2014). Donor-specific antibodies in allograft recipients: etiology, impact and therapeutic approaches. Curr Opin Organ Transplant.

[6] Del Bello A, Congy-Jolivet N, Muscari F, Lavayssiere L, Esposito L, Cardeau-Desangles I (2014). Prevalence, incidence and risk factors for donor-specific anti-HLA antibodies in maintenance liver transplant patients. Am J Transplant Off J Am Soc Transplant Am Soc Transplant Surg.

[7] Rashidi-Alavijeh J, Heinold A, Willuweit K, Baba HA, Horn PA, Paul A (2016). Diagnostics and treatment of a severe humoral rejection after liver transplantation: donor-specific antibodies as a still underestimated cause of graft failure. Z Gastroenterol.

[8] Del Bello A, Congy-Jolivet N, Danjoux M, Muscari F, Kamar N (2016). Donor-specific antibodies and liver transplantation. Hum Immunol.

[9] Ueno T, Zenitani M, Yamanaka H, Tanaka N, Uehara S, Tazuke Y (2016). Impact of donor-specific antibodies on graft fibrosis after pediatric living donor liver transplantation for biliary atresia. Transplant Proc.

[10] Susal C, Dohler B, Ruhenstroth A, Morath C, Slavcev A, Fehr T (2016). Donor-specific antibodies require preactivated immune system to harm renal transplant. EBioMedicine..

[11] Susal C, Zeier M (2018). Is there a need for additional DQ matching?. Clin J Am Soc Nephrol.

[12] Del Bello A, Congy-Jolivet N, Danjoux M, Muscari F, Lavayssiere L, Esposito L (2015). De novo donor-specific anti-HLA antibodies mediated rejection in liver-transplant patients. Transpl Int.

[13] Kaneku H, O'Leary JG, Banuelos N, Jennings LW, Susskind BM, Klintmalm GB (2013). De novo donor-specific HLA antibodies decrease patient and graft survival in liver transplant recipients. Am J Transplant.

[14] O'Leary JG, Demetris AJ, Philippe A, Freeman R, Cai J, Heidecke H (2017). Non-HLA antibodies impact on C4d staining, stellate cell activation and fibrosis in liver allografts. Transplantation..

[15] Musat AI, Agni RM, Wai PY, Pirsch JD, Lorentzen DF, Powell A (2011). The significance of donor-specific HLA antibodies in rejection and ductopenia development in ABO compatible liver transplantation. Am J Transplant Off J Am Soc Transplant Am Soc Transplant Surg.

[16] Oguma S, Belle S, Starzl TE, Demetris AJ (1989). A histometric analysis of chronically rejected human liver allografts: insights into the mechanisms of bile duct loss: direct immunologic and ischemic factors. Hepatology..

[17] Demetris AJ, Nakamura K, Yagihashi A, Iwaki Y, Takaya S, Hartman GG (1992). A clinicopathological study of human liver allograft recipients harboring preformed IgG lymphocytotoxic antibodies. Hepatology..

[18] Kozlowski T, Rubinas T, Nickeleit V, Woosley J, Schmitz J, Collins D (2011). Liver allograft antibody-mediated rejection with demonstration of sinusoidal C4d staining and circulating donor-specific antibodies. Liver Transpl.

[19] Steinhoff G, Wonigeit K, Pichlmayr R (1988). Analysis of sequential changes in major histocompatibility complex expression in human liver grafts after transplantation. Transplantation..

[20] Balan V, Ruppert K, Demetris AJ, Ledneva T, Duquesnoy RJ, Detre KM (2008). Long-term outcome of human leukocyte antigen mismatching in liver transplantation: results of the National Institute of Diabetes and Digestive and Kidney Diseases liver transplantation database. Hepatology..

[21] Ilyas JA, O'Mahony CA, Vierling JM (2011). Liver transplantation in autoimmune liver diseases. Best Pract Res Clin Gastroenterol.

[22] O'Leary JG, Kaneku H, Jennings LW, Banuelos N, Susskind BM, Terasaki PI (2013). Preformed class II donor-specific antibodies are associated with an increased risk of early rejection after liver transplantation. Liver Transpl.

[23] Gloor JM, Winters JL, Cornell LD, Fix LA, DeGoey SR, Knauer RM (2010). Baseline donor-specific antibody levels and outcomes in positive crossmatch kidney transplantation. Am J Transplant Off J Am Soc Transplant Am Soc Transplant Surg.

[24] Wozniak LJ, Hickey MJ, Venick RS, Vargas JH, Farmer DG, Busuttil RW (2015). Donor-specific HLA antibodies are associated with late allograft dysfunction after pediatric liver transplantation. Transplantation..

[25] O'Leary JG, Kaneku H, Demetris AJ, Marr JD, Shiller SM, Susskind BM (2014). Antibody-mediated rejection as a contributor to previously unexplained early liver allograft loss. Liver Transpl.

[26] Dar W, Agarwal A, Watkins C, Gebel HM, Bray RA, Kokko KE (2011). Donor-directed MHC class I antibody is preferentially cleared from sensitized recipients of combined liver/kidney transplants. Am J Transplant Off J Am Soc Transplant Am Soc Transplant Surg.

[27] Taner T, Gandhi MJ, Sanderson SO, Poterucha CR, De Goey SR, Stegall MD (2012). Prevalence, course and impact of HLA donor-specific antibodies in liver transplantation in the first year. Am J Transplant Off J Am Soc Transplant Am Soc Transplant Surg.

[28] Castillo-Rama M, Castro MJ, Bernardo I, Meneu-Diaz JC, Elola-Olaso AM, Calleja-Antolin SM (2008). Preformed antibodies detected by cytotoxic assay or multibead array decrease liver allograft survival: role of human leukocyte antigen compatibility. Liver Transpl.

